# Disease Burden and Progression in Patients with New-Onset Mild Cognitive Impairment and Alzheimer’s Disease Identified from Japanese Claims Data: Evidence from the LIFE Study

**DOI:** 10.3233/JAD-230471

**Published:** 2023-10-10

**Authors:** Haruhisa Fukuda, Hiroshi Kanzaki, Fumiko Murata, Megumi Maeda, Manabu Ikeda

**Affiliations:** a Department of Health Care Administration and Management, Kyushu University Graduate School of Medical Sciences, Fukuoka, Japan; b Department of Psychiatry, Osaka University Graduate School of Medicine, Suita, Japan

**Keywords:** Alzheimer’s disease, disease progression, mild cognitive impairment, real-world data

## Abstract

**Background:**

Accurate epidemiological data on mild cognitive impairment (MCI) and Alzheimer’s disease (AD) can inform the development of prevention and control measures, but there is a lack of such data in Japan.

**Objective:**

To investigate the disease burden and progression in patients with new-onset MCI or AD in Japan.

**Methods:**

Using claims data, this multi-region cohort study was conducted on new-onset MCI and AD patients in 17 municipalities from 2014 to 2021. To characterize the patients, we investigated their age, comorbidities, and long-term care (LTC) needs levels at disease onset according to region type (urban, suburban, or rural). Disease burden was examined using health care expenditures and LTC expenditures, which were estimated for 1, 2, and 3 years after disease onset. Kaplan-Meier curves were plotted for AD progression in new-onset MCI patients and death in new-onset AD patients.

**Results:**

We analyzed 3,391 MCI patients and 58,922 AD patients. In MCI and AD patients, health care expenditures were high in the first year ($13,035 and $15,858, respectively), but had declined by the third year ($8,278 and $10,414, respectively). In contrast, LTC expenditures (daily living support) steadily increased over the 3-year period (MCI patients: $1,767 to $3,712, AD patients: $6,932 to $9,484). In the third year after disease onset, 30.9% of MCI patients developed AD and 23.3% of AD patients had died.

**Conclusions:**

This provides an important first look at the disease burden and progression of MCI and AD in Japan, which are high-priority diseases for a rapidly aging population.

## INTRODUCTION

Population aging has been accompanied by an increase in many age-related health problems, as exemplified by the rising global prevalence of dementia [1]. Alzheimer’s disease (AD) accounts for approximately 60% of all dementia cases [2] and was estimated to affect 55 million people worldwide in 2021 [3]. Furthermore, the number of AD patients is projected to reach 78 million by 2030 [3]. In addition to imposing heavy clinical and social burdens on the lives of patients and their families, AD can cause substantial increases in health care expenditures and long-term care (LTC) expenditures [4, 5].

Due to the growing recognition of AD’s clinical and socioeconomic impact, there is an increasing emphasis on providing early treatment to persons with mild cognitive impairment (MCI) prior to AD onset. MCI refers to the stage between normal cognitive function and dementia, and is characterized by deficits in memory, attention, and other cognitive abilities that do not unduly affect an individual’s autonomy in daily life [6, 7]. Studies have estimated that 10–20% of older persons aged ≥65 years have MCI, and that 5–15% of these cases progress to dementia annually [8–11].

As one of the world’s most rapidly aging countries, Japan is particularly susceptible to the increasing prevalence of dementia and its associated burdens. In response to this problem, the Japanese government released the “Outline for the Promotion of Dementia Prevention Measures” in 2019, which aim to delay the onset of dementia in at-risk individuals and establish a society in which those with dementia can still be optimistic about daily life [12]. In order to design and implement effective measures, there is a need to accurately ascertain the patient profile and disease burden of individuals with MCI and AD. Municipal governments are already constantly engaged with a wide variety of health policy issues and require objective data to guide their decisions on allocating public funds to dementia-related measures. Although studies have been conducted using AD-related data [13, 14], there is a lack of basic epidemiological data on disease burden and disease progression for both MCI and AD in Japan.

The aim of this study was to provide a descriptive analysis of the disease burden and progression over 3 years in patients with new-onset MCI and AD in Japan. For this study, disease burden refers to health care expenditures and LTC expenditures, and disease progression refers to disease stage transitions (MCI to AD and AD to death). This study also examined the factors associated with disease stage transitions in MCI and AD patients. Disease burden and progression were analyzed using insurance claims data, which enable patient-level tracking of all health care and LTC services used in the real-world clinical setting.

## METHODS

### Database

This retrospective cohort study was conducted using data provided by the Longevity Improvement & Fair Evidence (LIFE) Study, which is a population-based multi-region database project created and administered by Kyushu University (Fukuoka, Japan) [15]. The LIFE Study is conducted through individual agreements with participating municipalities and involves the centralized collection of various health-related data types (such as health care claims data and LTC claims data) for research. Our study database included data from National Health Insurance enrollees aged 0–74 years and Latter-Stage Older Persons Health Care System enrollees aged ≥75 years residing in 17 municipalities across 4 prefectures. Data from different municipalities were standardized using a common data model before analysis [15]. In Japan, approximately 76% of all residents aged 70–74 years are enrolled in the National Health Insurance System [16], and 100% of all residents aged ≥75 years are enrolled in the Latter-Stage Older Persons Health Care System. As our study database comprised data from these insurance systems, the participants can be considered to be fairly representative of the municipalities’ resident populations.

### Participants

The study participants comprised patients with new-onset MCI or AD during the study period. While the study periods varied among the participating municipalities (depending on when they joined the LIFE Study), data were generally obtained from April 2014 to July 2021. The LIFE Study collects and maintains claims data for all insurance-covered clinical encounters (inpatient and outpatient) of insurance enrollees residing within the participating municipalities. During such encounters, patients are examined by physicians, and all physician-confirmed diagnoses are recorded in the claims data. Therefore, all recorded diagnoses in the claims data reflect actual diagnoses made by physicians in the clinical setting. MCI and AD were identified using the International Classification of Diseases, 10th Revision (ICD-10) codes recorded in each patient’s health care claims data (MCI: F067, AD: G30). For this study, we assumed that MCI and AD were diagnosed in accordance with national guidelines [17]. In Japan’s health care claims data, suspected diagnoses can be included to justify diagnostic tests or investigative procedures, and such diagnoses are marked with a “suspected” tag. As we aimed to focus on patients with MCI or AD, our analysis did not include cases with suspected diagnoses.

In each patient, the month that included the first recorded diagnosis of MCI or AD in the health care claims data during the study period was designated the “onset month”. However, some patients may have received earlier diagnoses of MCI or AD prior to their study period. For example, a patient whose AD onset month was June 2015 but had observation data from April 2015 onward would only have 2 months of observation before the onset month; if that patient had a previous diagnosis of AD in March 2015, it would be inaccurate to designate this case as “new-onset AD” in June 2015. Therefore, we excluded patients (1,028 from the MCI group and 57,106 from the AD group) without at least 6 months of observation before the onset month. Patients with ≤2 MCI records during the follow-up period were regarded as being re-diagnosed as not having MCI after further testing.

### Region type

The study was conducted using data from 17 municipalities. To account for the inherent regional differences in population density and infrastructure, the municipalities were categorized into 3 types: urban, suburban, and rural. Municipalities that are special wards of Tokyo or have a population of ≥300,000 were categorized as “urban municipalities” (4 municipalities). Municipalities that are adjacent to urban municipalities were categorized as “suburban municipalities” (7 municipalities). All other municipalities were categorized as “rural municipalities” (6 municipalities).

### Patient characteristics

We investigated patient sex, age, comorbidities, and LTC needs levels at MCI or AD onset according to region type. Age was categorized into 5 groups (≤64, 65–74, 75–84, 85–94, and ≥95 years). Comorbidities were identified from the recorded diagnoses in the health care claims data using ICD-10 codes. We calculated the Charlson Comorbidity Index [18, 19], and also identified records of hypertension, diabetes, mood disorder, arthrosis, fracture, coronary artery disease, heart failure, cerebrovascular disease, and malignant neoplasm within the 6-month period before MCI or AD onset. The diagnostic codes of these comorbidities are listed in Supplementary Table 1.

Using records from the LTC claims data, we identified each patient’s LTC needs level at MCI or AD onset. Under Japan’s LTC Insurance system, enrollees are assigned certified LTC needs levels (support needs levels 1–2 and LTC needs levels 1–5) according to their degree of physical and/or cognitive impairment (Supplementary Table 2). LTC needs certification is required to use LTC services (e.g., home-visit nursing services, rehabilitation services, and facility-based services), and persons with higher LTC needs levels are eligible for a wider range of services. Residents who wish to use LTC services under this system must first apply for certification at their local municipal office. A certification committee consisting of municipal government staff and healthcare experts determines each applicant’s LTC needs level based on home visits, family interviews, and written recommendations from physicians who are treating the applicant. LTC needs levels are designated using predetermined and standardized criteria in each municipality. For this study, we used each patient’s highest certified level during the follow-up period. Patients without any LTC claims data were categorized as “independent”. If patients had LTC needs certification but did not use any LTC services (and therefore did not generate any LTC claims), they were regarded as being independent.

For new-onset MCI patients, we calculated the duration from MCI onset until AD onset (the MCI onset month was treated as a full month in this estimate).

### Expenditures

We conducted a follow-up analysis of patients for 1 year (1-year cohort), 2 years (2-year cohort), and 3 years (3-year cohort) after MCI or AD onset. To estimate disease burden, we descriptively analyzed the health care expenditures and LTC expenditures for each cohort.

Health care expenditures referred to all medical-related expenditures for each patient and were not limited to the expenditures for the diagnosis and treatment of MCI or dementia. Similarly, LTC expenditures referred to all LTC-related expenditures for each patient. These expenditures were calculated based on each patient’s total payments (i.e., both insurer reimbursements and patient out-of-pocket copayments) for the use of medical care services and LTC services as recorded in the claims data. Japanese yen were converted to US dollars using the purchasing power parity rate in 2021 ($1.00 = 100.412 yen).

As a supplemental analysis, we also analyzed the LTC needs levels, health care facility use (outpatient visits, hospitalization duration, and use of testing services), and anti-AD drug prescriptions for each cohort. The details of these variables are provided in Supplementary Table 3.

### Statistical analysis

#### Disease burden and progression after MCI onset

We analyzed patients for 1 year, 2 years, and 3 years after MCI onset, with each cohort limited to patients with data in these follow-up durations. First, we conducted a descriptive analysis of health care expenditures and LTC expenditures for each cohort. As there may be substantial increases in health care expenditures and LTC expenditures just before death that are unrelated to disease progression, we excluded patients who had died during the study period in order to focus on expenditures attributable to MCI.

We then assessed the occurrence of AD and death in MCI patients in these cohorts. For this analysis, patients were classified as follows: MCI only; MCI and AD (without death); MCI and death (without AD); MCI, AD, and death; and AD before or during the same month as MCI onset. As our database did not include death certificate records, mortality was identified based on the recorded outcomes in the health care claims data. While the data include records on death, these are input by hospital staff and are not derived from death certificates.

Next, we plotted Kaplan–Meier curves to estimate the number of months from MCI onset until AD onset. These curves were plotted for the municipalities according to region type, age, sex, and number of comorbidities. Patients were censored at death.

#### Disease burden and progression after AD onset

We analyzed patients for 1 year, 2 years, and 3 years after AD onset, with each cohort limited to patients with data in these follow-up durations. First, we conducted a descriptive analysis of mortality, health care expenditures, and LTC expenditures for each cohort. All variables were assessed using the same methods as for the MCI patients. Similar to the calculation of expenditures in MCI patients, we excluded patients from each cohort who had died during the study period.

Next, we plotted Kaplan–Meier curves to estimate the number of months from AD onset until death. These curves were plotted for the municipalities according to region type, age, sex, and number of comorbidities.

#### Factors associated with progression from MCI to AD and from AD to death

Using Cox proportional hazards models, we conducted time-to-event (AD onset or death) analyses to identify the factors associated with progression from MCI to AD and from AD to death. The candidate factors included age, sex, comorbidities, LTC needs levels, and region type. LTC needs levels were categorized into low (support needs level 1–LTC needs level 1), moderate (LTC needs levels 2–3), and severe (LTC needs levels 4–5). For the analysis of progression from MCI to AD, patients were censored at death or at the end of the study period. For the analysis of progression from AD to death, patients were censored at the end of the study period. We calculated the hazard ratios (HRs) and 95% confidence intervals (CIs) of each factor for progression from MCI to AD and from AD to death.

All statistical analyses were performed using R version 4.1.0 (R Foundation for Statistical Computing, Vienna, Austria). Statistical significance was set at *p* < 0.05.

### Ethical considerations

The study was approved by the Kyushu University Institutional Review Board for Clinical Research (Approval No. 2019-406).

## RESULTS

### Disease burden and progression after MCI onset

From the 17 participating municipalities, we identified 3,391 patients with new-onset MCI in the health care claims data. Table 1 summarizes their characteristics according to region type. Among these, 2,307 patients were residing in urban municipalities, 512 in suburban municipalities, and 572 in rural municipalities. In all 3 region types, women accounted for approximately 60% of the patients, and the mean age was approximately 80 years. At MCI onset, 67.1–67.8% of patients had hypertension and 71.2–80.7% of patients had no LTC needs certification (i.e., independent) across the region types.

**Table 1:** Characteristics of new-onset MCI patients according to region type

	Urban Municipalities	Suburban Municipalities	Rural Municipalities
	(*n* = 2,307)	(*n* = 512)	(*n* = 572)
Women	1465 (63.5%)	307 (60.0%)	356 (62.2%)
Mean age at MCI onset, y	80.16 (6.65)	79.85 (6.75)	80.13 (6.59)
Age group at MCI onset, y
≤64	31 (1.3%)	11 (2.1%)	10 (1.7%)
65–74	359 (15.6%)	82 (16.0%)	90 (15.7%)
75–84	1286 (55.7%)	295 (57.6%)	320 (55.9%)
85–94	613 (26.6%)	119 (23.2%)	148 (25.9%)
≥95	18 (0.8%)	5 (1.0%)	4 (0.7%)
Comorbidities at MCI onset
Charlson Comorbidity Index	2.05 (1.75)	1.91 (1.65)	1.90 (1.67)
Hypertension	1548 (67.1%)	347 (67.8%)	384 (67.1%)
Diabetes	235 (10.2%)	57 (11.1%)	54 (9.4%)
Mood disorder	342 (14.8%)	68 (13.3%)	77 (13.5%)
Arthrosis	706 (30.6%)	149 (29.1%)	161 (28.1%)
Fracture	240 (10.4%)	38 (7.4%)	54 (9.4%)
Coronary artery disease	522 (22.6%)	129 (25.2%)	148 (25.9%)
Heart failure	597 (25.9%)	91 (17.8%)	97 (17.0%)
Cerebrovascular disease	662 (28.7%)	201 (39.3%)	174 (30.4%)
Malignant neoplasm	361 (15.6%)	69 (13.5%)	90 (15.7%)
LTC needs level at MCI onset
Independent	1642 (71.2%)	413 (80.7%)	453 (79.2%)
Support needs level 1	152 (6.6%)	18 (3.5%)	23 (4.0%)
Support needs level 2	126 (5.5%)	25 (4.9%)	31 (5.4%)
LTC needs level 1	200 (8.7%)	34 (6.6%)	41 (7.2%)
LTC needs level 2	98 (4.2%)	13 (2.5%)	13 (2.3%)
LTC needs level 3	50 (2.2%)	9 (1.8%)	6 (1.0%)
LTC needs level 4	29 (1.3%)	0 (0.0%)	5 (0.9%)
LTC needs level 5	10 (0.4%)	0 (0.0%)	0 (0.0%)
Duration until AD onset^a^
Before MCI onset	519 (22.5%)	115 (22.5%)	117 (20.5%)
In the same month as MCI onset	320 (13.9%)	42 (8.2%)	64 (11.2%)
Within 3 months of MCI onset	259 (17.8%)	39 (11.0%)	52 (13.3%)
Within 6 months of MCI onset	341 (25.0%)	57 (17.4%)	73 (20.2%)
Within 12 months of MCI onset	389 (36.1%)	77 (27.1%)	106 (35.1%)
Within 24 months of MCI onset	275 (52.8%)	81 (45.5%)	96 (51.3%)
Within 36 months of MCI onset	108 (59.3%)	53 (57.0%)	60 (65.2%)

Values are presented as number of patients (%) or mean (standard deviation). ^a^Only includes patients who could be observed for the stipulated follow-up periods or longer. AD, Alzheimer’s disease; LTC, long-term care; MCI, mild cognitive impairment.

**Fig. 1:**
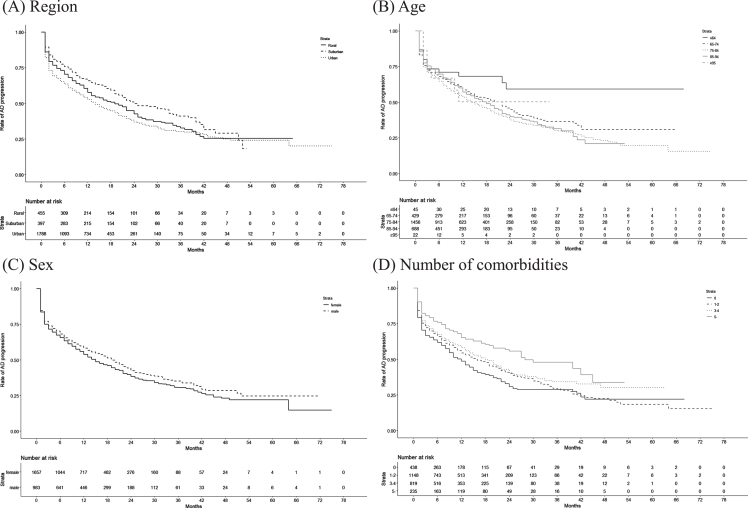
Kaplan–Meier curves of the duration from MCI onset until AD onset according to patient characteristics.

Table 2 shows the disease and mortality patterns in the new-onset MCI patients in the 1-year cohort (*n* = 3,653), 2-year cohort (*n* = 1,895), and 3-year cohort (*n* = 803). The proportions of patients with MCI and AD (without death) were 14.5% in the 1-year cohort, 23.1% in the 2-year cohort, and 27.3% in the 3-year cohort. The proportions of MCI patients without AD who died were 1.1% in the 1-year cohort, 2.0% in the 2-year cohort, and 2.6% in the 3-year cohort. Approximately 20% of new-onset MCI patients had AD before or during the same month as MCI in all 3 cohorts. The Kaplan–Meier curves of AD onset in MCI patients according to region type, age, sex, and number of comorbidities are shown in Fig. 1. The AD progression rates among new-onset MCI patients were 15.0% in the 1-year cohort, 24.7% in the 2-year cohort, and 30.9% in the 3-year cohort.

Table 3 presents the health care expenditures and LTC expenditures for patients with MCI only and patients with MCI and AD. Among the patients with MCI only, health care expenditures were high in the first year, but declined thereafter. In the 3-year cohort, the annual health care expenditures per MCI patient were $13,035 (standard error: $1,971) in the first year, $9,859 ($1,232) in the second year, and $8,278 ($1,018) in the third year. In contrast, LTC expenditures gradually increased over 3 years of follow-up. In the 3-year cohort, the annual LTC expenditures for daily living support per MCI patient were $1,767 (standard error: $414) in the first year, $2,755 ($521) in the second year, and $3,712 ($654) in the third year. The LTC needs levels, health care facility use per year, and anti-AD drug prescriptions in each cohort are presented in Supplementary Table 3.

**Table 2:** Disease and mortality patterns in new-onset MCI patients

Disease and mortality patterns	1-year cohort	2-year cohort	3-year cohort
	(*n* = 3,653)	(*n* = 1,895)	(*n* = 803)
No MCI (≤2 MCI records)^a^	1197 (32.8%)	549 (29.0%)	213 (26.5%)
MCI only	1127 (30.9%)	447 (23.6%)	148 (18.4%)
MCI and AD (without death)	531 (14.5%)	437 (23.1%)	219 (27.3%)
MCI and death (without AD)	41 (1.1%)	37 (2.0%)	21 (2.6%)
MCI, AD, and death	13 (0.4%)	26 (1.4%)	19 (2.4%)
AD before or during the same month as MCI onset	744 (20.4%)	399 (21.1%)	183 (22.8%)

Values are presented as number of patients (%). ^a^These patients were not included in Table 1 or Fig. 1. AD, Alzheimer’s disease; MCI, mild cognitive impairment.

**Table 3:** Expenditures after MCI onset in the 1-year, 2-year, and 3-year cohorts

	MCI only			MCI and AD (without death)		
	1-year cohort	2-year cohort	3-year cohort	1-year cohort	2-year cohort	3-year cohort
	(*n* = 1,127)	(*n* = 447)	(*n* = 148)	(*n* = 531)	(*n* = 437)	(*n* = 219)
Health care expenditures, US$
First year	11987 [538]	12528 [960]	13035 [1971]	11939 [713]	10751 [696]	11025 [1014]
Second year	–	9654 [721]	9859 [1232]		11862 [762]	9788 [857]
Third year	–	–	8278 [1018]			11612 [1209]
LTC expenditures, US$
First year (Medical component)	627 [64]	463 [86]	183 [91]	608 [82]	510 [89]	510 [121]
Second year (Medical component)	–	682 [110]	428 [142]		882 [123]	877 [190]
Third year (Medical component)	–	–	494 [145]			1231 [233]
First year (Daily living support component)	2621 [184]	2473 [309]	1767 [414]	2603 [224]	2429 [261]	2231 [363]
Second year (Daily living support component)	–	3405 [378]	2755 [521]		5223 [395]	4277 [529]
Third year (Daily living support component)	–	–	3712 [654]			7025 [632]
Health care &LTC expenditures, US$
First year	15235 [604]	15464 [1057]	14985 [2037]	15149 [808]	13691 [814]	13766 [1187]
Second year	–	13741 [876]	13042 [1486]		17967 [890]	14942 [1031]
Third year	–	–	12483 [1347]			19868 [1397]

Values are presented as number of patients (%) or mean [standard error]. AD, Alzheimer’s disease; LTC, long-term care; MCI, mild cognitive impairment.

### Disease burden and progression after AD onset

From the 17 participating municipalities, we identified 58,922 patients with new-onset AD in the health care claims data. Table 4 summarizes their characteristics according to region type. Among these, 41,227 patients were residing in urban municipalities, 7,922 in suburban municipalities, and 9,773 in rural municipalities. In all 3 region types, women accounted for approximately 65% of the patients, and the mean age was approximately 83 years. At AD onset, 57.8–63.3% of patients had hypertension and 8.3–9.8% of patients had diabetes across the region types. The certified care needs levels varied among the region types: 55.2%, 63.4%, and 58.7% of patients had no LTC needs certification in the urban, suburban, and rural municipalities, respectively.

**Table 4:** Characteristics of new-onset AD patients according to region type

	Urban Municipalities	Suburban Municipalities	Rural Municipalities
	(*n* = 41,227)	(*n* = 7,922)	(*n* = 9,773)
Women	26309 (63.8%)	5135 (64.8%)	6504 (66.6%)
Mean age at AD onset, y	82.73 (7.25)	82.33 (7.30)	83.23 (7.33)
Age group at AD onset, y
≤64	492 (1.2%)	91 (1.1%)	110 (1.1%)
65–74	4210 (10.2%)	986 (12.4%)	1005 (10.4%)
75–84	19251 (46.7%)	3651 (46.1%)	4168 (42.6%)
85–94	15640 (37.9%)	2905 (36.7%)	4056 (41.4%)
≥95	1634 (4.0%)	289 (3.6%)	434 (4.4%)
Comorbidities at AD onset
Charlson Comorbidity Index	1.88 (1.76)	1.62 (1.65)	1.70 (1.62)
Hypertension	26102 (63.3%)	4582 (57.8%)	5987 (61.3%)
Diabetes	4032 (9.8%)	700 (8.8%)	809 (8.3%)
Mood disorder	5023 (12.2%)	928 (11.7%)	1242 (12.7%)
Arthrosis	10391 (25.2%)	1840 (23.2%)	2613 (26.7%)
Fracture	5307 (12.9%)	939 (11.9%)	1258 (12.9%)
Coronary artery disease	9157 (22.2%)	1702 (21.5%)	2409 (24.6%)
Heart failure	11547 (28.0%)	1502 (19.0%)	1930 (19.7%)
Cerebrovascular disease	11546 (28.0%)	2542 (32.1%)	3073 (31.4%)
Malignant neoplasm	5621 (13.6%)	976 (12.3%)	1245 (12.7%)
LTC needs level at AD onset
Independent	22742 (55.2%)	5026 (63.4%)	5727 (58.7%)
Support needs level 1	1770 (4.3%)	338 (4.3%)	344 (3.5%)
Support needs level 2	1902 (4.6%)	336 (4.2%)	423 (4.3%)
LTC needs level 1	4855 (11.8%)	735 (9.3%)	1177 (12.0%)
LTC needs level 2	3617 (8.8%)	562 (7.1%)	804 (8.2%)
LTC needs level 3	2685 (6.5%)	386 (4.9%)	543 (5.6%)
LTC needs level 4	2269 (5.5%)	354 (4.5%)	483 (4.9%)
LTC needs level 5	1387 (3.4%)	185 (2.3%)	273 (2.8%)

Values are presented as number of patients (%) or mean (standard deviation). AD, Alzheimer’s disease; LTC, long-term care.

**Fig. 2:**
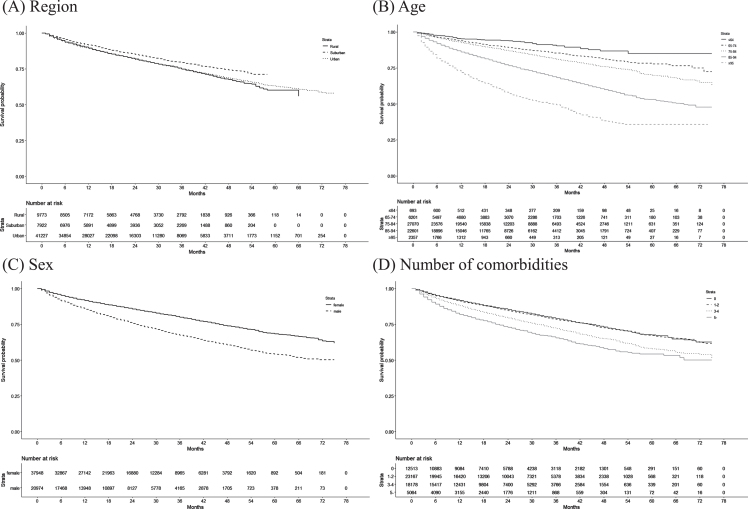
Kaplan–Meier curves of the duration from AD onset until death according to patient characteristics.

Table 5 shows the mortality, health care expenditures, and LTC expenditures for the new-onset AD patients in the 1-year cohort (*n* = 45,552), 2-year cohort (*n* = 30,158), and 3-year cohort (*n* = 17,006). The proportions of AD patients who died were 10.4% in the 1-year cohort, 17.7% in the 2-year cohort, and 23.3% in the 3-year cohort. The Kaplan–Meier curves of death in AD patients according to region type, age, sex, and number of comorbidities are shown in Fig. 2. Health care expenditures were high in the first year, but declined thereafter. In the 3-year cohort, the annual health care expenditures per AD patient were $15,858 (standard error: $148) in the first year, $11,270 ($130) in the second year, and $10,414 ($134) in the third year. However, the daily living support component of LTC expenditures generally increased after the first year. In the 3-year cohort, the annual LTC expenditures for daily living support per AD patient were $6,932 (standard error: $86) in the first year, $8,666 ($98) in the second year, and $9,484 ($102) in the third year. The LTC needs levels, health care facility use per year, and anti-AD drug prescriptions in each cohort are presented in Supplementary Table 4.

**Table 5:** Mortality and expenditures after AD onset in the 1-year, 2-year, and 3-year cohorts

	1-year cohort	2-year cohort	3-year cohort
	(*n* = 45,552)	(*n* = 30,158)	(*n* = 17,006)
Death	4755 (10.4%)	5337 (17.7%)	3968 (23.3%)
Health care expenditures, US$
First year	15912 [92]	15966 [113]	15858 [148]
Second year	–	11287 [98]	11270 [130]
Third year	–	–	10414 [134]
LTC expenditures, US$
First year (Medical component)	924 [13]	874 [15]	792 [20]
Second year (Medical component)	–	947 [17]	877 [22]
Third year (Medical component)	–	–	910 [23]
First year (Daily living support component)	7885 [54]	7625 [66]	6932 [86]
Second year (Daily living support component)	–	9112 [75]	8666 [98]
Third year (Daily living support component)	–	–	9484 [102]
Health care &LTC expenditures, US$
First year	24721 [108]	24465 [131]	23582 [171]
Second year	–	21346 [125]	20813 [165]
Third year	–	–	20808 [171]

Values are presented as number of patients (%) or mean [standard error]. AD, Alzheimer’s disease; LTC, long-term care.

### Factors associated with progression from MCI to AD and from AD to death

Table 6 presents the HRs of the factors associated with progression from MCI to AD and from AD to death. When compared with MCI patients in rural municipalities, those in urban municipalities had a significantly higher hazard of AD onset (HR: 1.21, 95% CI: 1.05–1.39), whereas those in suburban municipalities had a significantly lower hazard of AD onset (0.81, 0.67–0.99). Furthermore, women and increasing age were significantly associated with progression from MCI to AD. In contrast, MCI patients with higher LTC needs levels had a significantly lower hazard of AD onset than independent patients.

When compared with AD patients in rural municipalities, those in suburban municipalities had a significantly lower hazard of mortality (HR: 0.83, 95% CI: 0.77–0.89), but those in urban municipalities did not show a significant association with mortality (0.95, 0.91–1.00). Women, hypertension, and arthrosis had significantly lower hazards of mortality. However, increasing age, diabetes, fracture, heart failure, cerebrovascular disease, malignant neoplasm, and higher LTC needs levels had significantly higher hazards of mortality.

**Table 6:** Factors associated with progression from MCI to AD and from AD to death

	MCI to AD		AD to death	
	HR	95% CI	HR	95% CI
Women	1.13	1.01–1.27	0.50	0.48–0.52
Age	1.01	1.00–1.02	1.06	1.05–1.06
Region type
Rural	REF		REF
Suburban	0.81	0.67–0.99	0.83	0.77–0.89
Urban	1.21	1.05–1.39	0.95	0.91–1.00
Comorbidities
Hypertension	1.03	0.92–1.16	0.9	0.86–0.94
Diabetes	0.99	0.83–1.18	1.29	1.21–1.37
Mood disorder	0.86	0.73–1.02	1.05	0.99–1.11
Arthrosis	0.92	0.81–1.03	0.79	0.76–0.83
Fracture	0.78	0.64–0.96	1.12	1.06–1.18
Coronary artery disease	0.97	0.84–1.10	0.96	0.91–1.00
Heart failure	0.91	0.79–1.05	1.47	1.40–1.53
Cerebrovascular disease	0.89	0.78–1.01	1.05	1.01–1.09
Malignant neoplasm	0.88	0.76–1.03	1.62	1.54–1.70
LTC needs level^a^
Independent	REF		REF
Low level	0.84	0.72–0.98	1.20	1.13–1.26
Moderate level	0.71	0.54–0.95	1.90	1.80–2.00
Severe level	0.22	0.08–0.58	3.13	2.95–3.31

^a^Low level included support needs level 1–LTC needs level 1, moderate level included LTC needs levels 2–3, and severe level included LTC needs levels 4–5. AD, Alzheimer’s disease; CI, confidence interval; HR, hazard ratio; LTC, long-term care; MCI, mild cognitive impairment.

## DISCUSSION

Using a LIFE Study database comprising health care claims data and LTC claims data from 17 municipalities, we examined the disease burden and progression in MCI and AD patients for the first time in Japan. This study characterized the patients at MCI and AD onset, and shed light on the disease stage transitions from MCI to AD as well as from AD to death. The categorization of the 17 municipalities into urban, suburban, and rural types revealed region-associated differences in the patient profiles.

Our study estimated the AD progression rate among new-onset MCI patients to be 15.0% in the 1-year cohort, 24.7% in the 2-year cohort, and 30.9% in the 3-year cohort. While the AD progression rate was fairly high in the first 2 years, the increase in the third year was less precipitous. These estimates were generally higher than those of previous studies. For example, Shimada et al. estimated the annual AD progression rate to be 3.4% among 743 community-dwelling older Japanese persons with MCI [14]. Population-based studies from other countries have reported AD progression rates to be 10–15%, with estimates that extended both below and above our measurements [20]. For example, Artero et al. and Farias et al. reported low AD progression rates [21, 22]. Artero et al. estimated the AD progression rate to be 6.6% after 4 years among 1,879 community-dwelling MCI patients in France [21]. Farias et al. followed-up 60 community-dwelling MCI patients in the US for a mean duration of 2.4 years and estimated the annual AD progression rate to be only 3% [22]. Next, Peters et al. and Manly et al. reported AD progression rates that were similar to our measurements [23, 24]. Peters et al. followed-up 230 community-dwelling MCI patients in the US for a mean duration of 3.3 years and estimated the annual AD progression rate to be 12% [23]. Similarly, Manly et al. monitored 1,800 community-dwelling MCI patients in the US, and estimated the overall annual AD progression rate to be 9.7% for those aged 75–79% and 11.1% for those aged ≥80 years [24]. While our estimated AD progression rates were within the range reported by previous studies, they were higher than those of others. A possible explanation for this discrepancy is that our participants may have included a large proportion of those with more progressive subjective symptoms and had therefore consulted with a physician at a health care facility. In contrast, other studies may have included MCI cases that were only identified using general community-based testing for residents, which could result in a high proportion of early-stage MCI cases that had just progressed from the preclinical to prodromal stages. Accordingly, it is possible that our target MCI patients, all of whom had clinical diagnoses, had more severe symptoms and were more susceptible to AD progression than those of other studies.

In our analysis, we found that a substantial proportion of new-onset MCI patients were also diagnosed with AD before or during the same month as their MCI diagnosis. This suggests that the timing of clinical visits may affect the accuracy and timeliness of monitoring these diseases using claims data. These cases could be the result of physicians initially underestimating the extent of cognitive decline, resulting in an MCI diagnosis being quickly followed by an AD diagnosis. In addition, patients already diagnosed with AD may have been subsequently diagnosed with MCI at a different health care facility. Further studies are needed to determine the underlying reasons for these observations. Next, we noted that health care expenditures were highest in the year in which MCI or AD were diagnosed. This may indicate that many patients were diagnosed with these conditions while being treated for other diseases, which would have added to their total annual expenditures. In other words, cognitive decline may only have been tested and diagnosed in these patients during clinical encounters for other diseases. Previous registry-based studies in other countries have estimated MCI prevalence to be 16–38% [20,25,26]. In contrast, our estimated MCI prevalence was much lower at only 0.49% (data not shown), which suggests that a large number of individuals with MCI in Japan have not been formally diagnosed. This implies that primary care physicians in Japan remain inexperienced in recognizing and diagnosing this condition, and that there is a lack of community-based testing. We can infer from these results that despite universal health coverage and widespread access to health care, there are many undiagnosed cases of MCI within the Japanese population.

Our study also characterized the disease burden in patients from an economic perspective. Although previous studies have estimated the health care expenditures of AD patients [27, 28], few have included LTC expenditures [5]. Our study therefore provides valuable insight into the annual health care expenditures and LTC expenditures for the first, second, and third years after AD onset. In our search of the literature, we found only 5 studies that had estimated the health care expenditures associated with MCI [27, 29–32]. We calculated the mean health care expenditures to be $13,035 per patient in the first year after MCI onset, which fell into the range reported by previous studies [27, 29–32]. Leibson et al., which employed a similar study design to ours, estimated the annual expenditures to be $6,784 per patient among 537 MCI patients in the US [30]. Moreover, we estimated the mean LTC expenditures for daily living support to be $1,753 per patient in the first year after MCI onset. To our knowledge, this is the first study to quantify the LTC expenditures incurred by MCI patients. Our estimated health care expenditures in AD patients ($15,858 in the first year, $11,270 in the second year, and $10,414 in the third year) were generally lower than those reported in a US study ($23,917 in the first year, $16,286 in the second year, and $12,766 in the third year) [28]. Nevertheless, both studies also found that health care expenditures decreased after the first year. In Leibson et al., the estimated medical costs of newly discovered dementia were approximately 1.39 times that of MCI [30]. This was similar to our study, in which the first-year expenditures of AD patients were 1.22 times that of MCI patients. We calculated the 95% CIs for annual expenditures to be $9,171–$16,899 for MCI patients and $15,568–$16,148 for AD patients; as these CIs overlapped, there was no significant difference in estimates.

Our multi-region analysis of 17 municipalities categorized according to region type produced 2 major findings. First, progression from MCI to AD was higher in urban municipalities than in rural municipalities (HR: 1.21, *P* = 0.007). To our knowledge, no studies have explored the regional differences in the progression from MCI to AD. We posit that our observed higher AD progression rates in urban municipalities may be indicative of regional differences in medical infrastructure, such as urban areas having more specialists that can recognize the progression from MCI to AD. Second, mortality in AD patients was lower in suburban municipalities than in rural municipalities (HR: 0.82, *P* < 0.001). This was similar to a previous study from the US [33]. In our analysis, suburban municipalities had a higher proportion of functionally independent AD patients. Nevertheless, mortality was still lower in suburban municipalities even after adjusting for variations in LTC needs levels. As it was difficult to explain this observation in our sample of 17 municipalities, further studies that include a larger number of municipalities are needed to clarify this relationship.

While the LIFE Study is designed to produce real-world evidence to support the evaluation and improvement of health-related problems through research, there are limitations that must be considered in the interpretation of our findings. First, we assumed that MCI and AD were diagnosed based on uniform criteria in accordance with Japan’s national guidelines. However, our retrospective study used recorded diagnoses from claims data, and we could not account for any physician-level variations. In particular, MCI diagnoses may be dependent on the experience and expertise of each diagnosing physician. As shown in Table 2, approximately 20% of the MCI patients had been diagnosed with AD before or during the MCI diagnosis. It is possible that patients who should have been diagnosed with MCI were instead diagnosed with AD. Although not shown in our results, the increase in MCI patients was more prominent than the increase in AD patients over time. This suggests that physicians’ techniques and expertise in diagnosing MCI are still improving. Second, our study only included patients with confirmed diagnoses of MCI or AD, which may have introduced selection bias into the analysis. This may also have omitted patients with relatively mild symptoms, which could have led to overestimating the AD progression rates from MCI. Furthermore, our study did not include patients who had sought care under private insurance. However, the number of such cases is unlikely to be large due to the extensive coverage of public insurance among older persons in Japan. Third, our study did not include other types of dementia, such as vascular dementia. In addition, our analyses did not take into account the different subtypes of MCI (e.g., amnestic versus non-amnestic and multi-domain versus single-domain) due to data limitations. Amnestic MCI is associated with faster progression to dementia [34], and our findings should be interpreted with consideration to this limitation. Fourth, our unadjusted mean estimates of health care expenditures and LTC expenditures were not those directly attributable to MCI or AD, but were all-cause expenditures incurred by patients with these conditions. As such, these estimates included expenditures for other diseases. Moreover, the estimates were obtained from patients who could be followed-up for 1–3 years and did not include those who had died during the study period. Patients who had died during the study period were censored but were not accounted for using statistical methods [35]. Also, these estimates did not include unpaid informal care provided by family members. Furthermore, health-related expenditure data tend to be heavily right-skewed, and a small proportion of high-cost cases can inordinately increase mean estimates. Therefore, caution must be exercised when comparing our estimates with those of other studies [28, 36]. Fifth, our data did not include information on education levels, socioeconomic status, or lifestyle habits. As a consequence, we could not account for variations in these factors.

Despite these limitations, our study provides an important first look at the disease burden and progression in patients with new-onset MCI and AD in Japan. As Japan is one of the few developed countries with an increasing dementia prevalence [37], there is a need to further deepen our understanding of the characteristics of MCI and AD patients, and to devise appropriate prevention and control measures based on epidemiological data. Our results may contribute to the formulation of such measures and guide the appropriate allocation of resources commensurate with the burden of these diseases.

## Supplementary Material

Supplementary Material

## Data Availability

The data used in this study were acquired under agreements between Kyushu University and the participating municipalities, which stipulate that the data can only be used by authorized research institutions and cannot be shared with third parties.
