# Supplementary material for: Disease Burden and Progression in Patients with New-Onset Mild Cognitive Impairment and Alzheimer’s Disease Identified from Japanese Claims Data: Evidence from the LIFE Study
Source: J Alzheimers Dis. 2023 Oct 10;95(4):1559–72. doi: 10.3233/JAD-230471 (PMC10578250; doi:10.3233/JAD-230471)
Supplement: Supplementary Material [file jad-95-jad230471-s001.pdf]

Supplementary Material

Disease Burden and Progression in Patients with New-Onset Mild Cognitive Impairment and Alzheimer's Disease Identified from Japanese Claims Data: Evidence from the LIFE Study

Supplementary Table 1. Diagnostic codes of comorbidities, procedural codes for health care facility use, and anti-AD drug prescription codes

Factors	Codes
Comorbidities	
Hypertension	I10, I11, I12, I13, I15
Diabetes	E10–E14
Mood disorder	F30–F39
Arthrosis	M15–M19
Fracture	S32, S42, S52, S62, S72, S82, S92, T02, T08, T10, T12
Coronary artery disease	I20–I25
Heart failure	I50
Cerebrovascular disease	I60–I69
Malignant neoplasm	C, D00-D09
Outpatient visits at health care facilities	
Fees for a first visit	111000110 or 111012510
Fees for a follow-up visit	112007410 or 112007950
Testing services	
Cognitive function tests	160085910 or 160085410
CT scans	170011810, 170028610, 170033410, 170034910, 170038710, 170038810, 170038910, 170039010,170039110
MRI scans	170015210, 170020110, 170033510, 170033750, 170035010, 170036270, 170037970, 170038530
SPECT scans	170015010
Anti-AD drugs	
Donepezil	1190012
Memantine	1190018
Galantamine	1190019
Rivastigmine	1190700

Comorbidities were identified using International Classification of Diseases, 10th Revision codes. Health care facility use and anti-AD drug use were identified using Japanese procedural and prescription codes. AD, Alzheimer's disease; CT, computed tomography; MRI, magnetic resonance imaging, SPECT, single-photon emission computed tomography.

Supplementary Table 2. Description of support needs levels and LTC needs levels used in Japan's LTC Insurance System

	**General description**
Support needs level 1	Subject is mostly able to independently perform basic activities of daily living, but requires some degree of support for instrumental activities of daily living.
Support needs level 2	Subject is less able to perform instrumental activities of daily living when compared to support needs level 1, and requires a greater degree of support.
LTC needs level 1	Subject finds it difficult to independently perform basic activities of daily living. Subject also is less able to perform instrumental activities of daily living when compared to support needs level 2, and requires partial LTC.
LTC needs level 2	In addition to the conditions described in LTC needs level 1, the subject also requires partial LTC to perform basic activities of daily living.
LTC needs level 3	Subject is less able to perform both basic and instrumental activities of daily living as compared to LTC needs level 2, and generally requires extensive LTC.
LTC needs level 4	In addition to the conditions described in LTC needs level 3, the subject has further reductions in functional ability and experiences difficulties in going about daily life without extensive LTC.
LTC needs level 5	Subject has further reductions in functional ability when compared to LTC needs level 4, and essentially cannot go about daily life without extensive LTC.

LTC, long-term care.

Supplementary Table 3. LTC needs levels, health care facility use, and anti-AD drug prescriptions after MCI onset in the 1-year, 2-year, and 3-year cohorts

	MCI only			MCI and AD (without death)		
	**1-year cohort (n = 1,127)**	**2-year cohort (n = 447)**	**3-year cohort (n = 148)**	**1-year cohort (n = 531)**	**2-year cohort (n = 437)**	**3-year cohort (n = 219)**
LTC needs levels (highest certified level during the study period)						
Independent	689 (61.1%)	272 (60.9%)	93 (62.9%)	280 (52.8%)	182 (41.7%)	67 (30.6%)
Support needs level 1	93 (8.3%)	27 (6.0%)	6 (4.1%)	30 (5.6%)	20 (4.6%)	6 (2.7%)
Support needs level 2	74 (6.6%)	27 (6.0%)	7 (4.7%)	31 (5.8%)	19 (4.3%)	7 (3.2%)
LTC needs level 1	139 (12.3%)	52 (11.6%)	14 (9.5%)	118 (22.2%)	118 (27.0%)	67 (30.6%)
LTC needs level 2	53 (4.7%)	32 (7.2%)	11 (7.4%)	40 (7.5%)	54 (12.4%)	38 (17.4%)
LTC needs level 3	35 (3.1%)	14 (3.1%)	7 (4.7%)	17 (3.2%)	24 (5.5%)	18 (8.2%)
LTC needs level 4	30 (2.7%)	14 (3.1%)	8 (5.4%)	12 (2.3%)	15 (3.4%)	12 (5.5%)
LTC needs level 5	14 (1.2%)	9 (2.0%)	2 (1.4%)	3 (0.6%)	5 (1.1%)	4 (1.8%)
Health care facility use						
No. of outpatient visits per year[1]	6.60 [0.41]	5.49 [0.70]	3.61 [0.77]	4.59 [0.64]	2.22 [0.23]	1.41 [0.22]
Hospitalization duration (days) per year[2]	20.07 [2.33]	17.21 [2.74]	15.49 [4.19]	32.46 [5.34]	24.84 [3.40]	25.14 [4.34]
No. of cognitive function tests per year[3]	0.65 [0.03]	0.29 [0.03]	0.13 [0.02]	0.70 [0.04]	0.30 [0.03]	0.12 [0.02]
No. of CT scans per year[3]	0.50 [0.03]	0.32 [0.03]	0.23 [0.04]	0.42 [0.04]	0.25 [0.02]	0.18 [0.03]
No. of MRI scans per year[3]	0.52 [0.02]	0.38 [0.02]	0.31 [0.03]	0.63 [0.03]	0.35 [0.02]	0.24 [0.02]
No. of SPECT scans per year[3]	0.10 [0.01]	0.08 [0.01]	0.05 [0.01]	0.18 [0.02]	0.11 [0.01]	0.08 [0.01]
Anti-AD drug prescriptions[4]						
None	1105 (98.0%)	438 (98.0%)	145 (98.0%)	101 (19.0%)	72 (16.5%)	28 (12.8%)
1 drug type	20 (1.8%)	7 (1.6%)	3 (2.0%)	354 (66.7%)	280 (64.1%)	119 (54.3%)
2 drug types	2 (0.2%)	2 (0.4%)	0 (0.0%)	71 (13.4%)	74 (16.9%)	52 (23.7%)
3 drug types	0 (0.0%)	0 (0.0%)	0 (0.0%)	5 (0.9%)	9 (2.1%)	18 (8.2%)
4 drug types	0 (0.0%)	0 (0.0%)	0 (0.0%)	0 (0.0%)	2 (0.5%)	2 (0.9%)

Values are presented as number of patients (%) or mean [standard error]. AD, Alzheimer's disease; CT, computed tomography; LTC, long-term care; MCI, mild cognitive impairment; MRI, magnetic resonance imaging; SPECT, single-photon emission computed tomography.

[1]The numbers of outpatient visits at health care facilities were calculated using Japanese procedural codes recorded in the health care claims data: fees for a first visit and fees for a follow-up visit.

[2]Hospitalization durations were estimated from the health care claims data.

[3]Testing services included cognitive function tests, CT scans, MRI scans, and SPECT scans.

[4]Anti-AD drug prescriptions were identified using prescription codes recorded in the health care claims data: donepezil, memantine, galantamine, and rivastigmine.

Supplementary Table 4. LTC needs levels, health care facility use, and anti-AD drug prescriptions after AD onset in the 1-year, 2-year, and 3-year cohorts

	**1-year cohort (n = 45,552)**	**2-year cohort (n = 30,158)**	**3-year cohort (n = 17,006)**
LTC needs levels (highest certified level during the study period)			
Independent	16185 (35.5%)	8849 (29.3%)	4294 (25.2%)
Support needs level 1	1526 (3.4%)	639 (2.1%)	300 (1.8%)
Support needs level 1	1280 (2.8%)	621 (2.1%)	269 (1.6%)
LTC needs level 1	8726 (19.2%)	5543 (18.4%)	2908 (17.1%)
LTC needs level 2	5498 (12.1%)	4128 (13.7%)	2488 (14.6%)
LTC needs level 3	4611 (10.1%)	3670 (12.2%)	2241 (13.2%)
LTC needs level 4	4620 (10.1%)	3774 (12.5%)	2501 (14.7%)
LTC needs level 5	3106 (6.8%)	2934 (9.7%)	2005 (11.8%)
Health care facility use			
No. of outpatient visits per year	11.02 [0.09]	9.96 [0.11]	9.37 [0.14]
Hospitalization duration (days) per year	43.66 [0.60]	34.04 [0.48]	30.65 [0.51]
No. of cognitive function tests per year	0.35 [0.00]	0.18 [0.00]	0.13 [0.00]
No. of CT scans per year	0.78 [0.01]	0.57 [001]	0.48 [0.01]
No. of MRI scans per year	0.39 [0.00]	0.26 [0.00]	0.22 [0.00]
No. of SPECT scans per year	0.07 [0.00]	0.04 [0.00]	0.02 [0.00]
Anti-AD drug prescriptions			
None	13545 (29.7%)	7745 (25.7%)	3914 (23.0%)
1 drug type	25783 (56.6%)	16476 (54.6%)	9000 (52.9%)
2 drug types	5519 (12.1%)	4998 (16.6%)	3308 (19.5%)
3 drug types	650 (1.4%)	844 (2.8%)	700 (4.1%)
4 drug types	55 (0.1%)	95 (0.3%)	84 (0.5%)

Values are presented as number of patients (%) or mean [standard error]. AD, Alzheimer's disease; CT, computed tomography; LTC, long-term care; MRI, magnetic resonance imaging; SPECT, single-photon emission computed tomography.
